# Genome Analysis of A Novel South African Cydia pomonella granulovirus (CpGV-SA) with Resistance-Breaking Potential

**DOI:** 10.3390/v11070658

**Published:** 2019-07-18

**Authors:** Boitumelo Motsoeneng, Michael D. Jukes, Caroline M. Knox, Martin P. Hill, Sean D. Moore

**Affiliations:** 1 Department of Biochemistry and Microbiology, Rhodes University, P.O. Box 94, Grahamstown 6140, South Africa; 2 Centre for Biological Control, Department of Zoology and Entomology, Rhodes University, P.O. Box 94, Grahamstown 6140, South Africa; 3 Citrus Research International, P.O. Box 5095, Walmer, Port Elizabeth 6065, South Africa

**Keywords:** Cydia pomonella granulovirus, codling moth, single nucleotide polymorphism (SNP), single nucleotide variation (SNV)

## Abstract

The complete genome of an endemic South African Cydia pomonella granulovirus isolate was sequenced and analyzed. Several missing or truncated open reading frames (ORFs) were identified, including a 24 bp deletion in the pe38 gene which is reported to be associated with type I resistance-breaking potential. Comparison of single nucleotide polymorphisms (SNPs) with five other fully sequenced CpGV isolates identified 67 unique events, 47 of which occurred within ORFs, leading to several amino acid changes. Further analysis of single nucleotide variations (SNVs) within CpGV-SA revealed that this isolate consists of mixed genotypes. Phylogenetic analysis using complete genome sequences placed CpGV-SA basal to M, I12 and E2 and distal to S and I07 but with no distinct classification into any of the previously defined CpGV genogroups. These results suggest that CpGV-SA is a novel and genetically distinct isolate with significant potential as a biopesticide for management of codling moth (CM), not only in South Africa, but potentially in other pome fruit producing countries, particularly where CM resistance to CpGV has been reported.

## 1. Introduction

The codling moth, *Cydia pomonella* (CM; Lepidoptera: Tortricidae), is an economically important agricultural pest causing extensive damage to pome fruit production in most parts of the temperate world, including South Africa [1,2]. Among the options available for management of this pest is the Cydia pomonella granulovirus, CpGV, belonging to the dsDNA family *Baculoviridae* (genus *Betabaculovirus*), which is now registered in 34 countries worldwide as a highly selective, effective and environmentally safe biological control agent [3]. Since its discovery in Mexico in 1963 [4], several genetically distinct isolates of CpGV have been described from various regions of the world and have been classified into phylogenetic genome groups A–E. Representative examples of members of different genome groups include M (group A), E2 (group B), I07 (group C), I12 (group D) and S (group E) [5,6]. Currently, there are six full CpGV genome sequences from different geographic origins available on the NCBI database for comparison with novel isolates. Recently, these genomes have been compared to infer intra-species diversity and to create a genome-wide map of CpGV based on specific positions of single nucleotide polymorphisms (SNPs) within each genome [7].

Commercial CpGV products have been used since the late 1980s for the control of CM in pome fruit growing areas worldwide and, initially, most of them contained the Mexican isolate, CpGV-M, as the active ingredient [8]. In 2004 and 2005, resistance in CM field populations was reported in France and Germany for the first time [9,10] and subsequently confirmed by laboratory bioassays [11,12]. This mode of resistance, termed Type I [13], was found to be isolate-specific and directed only at CpGV-M (genogroup A). Furthermore, sequence analysis confirmed that it was associated with the presence of a 24 bp motif in the pe38 gene, which was missing in so-called “resistance-breaking” isolates that could overcome CM resistance [6,14,15,16]. The observation of widespread CpGV resistance prompted many investigations across Europe, leading to field trials and alternative CpGV commercial formulations for CM management [3,14,15,16,17,18,19,20]. Recently, infection studies and crossing experiments with laboratory CM strains revealed additional modes of resistance, namely, Type II directed against most known CpGV isolates except those belonging to genotype B [13] and a more complex Type III resistance, which appeared to target at least two CpGV genome groups, A and E [21]. These observations highlight the importance of further understanding the genetic basis for resistance and the co-evolution of virus and host if CpGV-based management is to be sustained as one of the most effective control measures in pome fruit production globally.

The South African pome fruit industry is a well-established, multi-billion Rand operation, cultivating apples and pears for both local consumption and export mainly to Europe, the Far East, Asia, and the rest of the African continent [22]. Despite its reputation for producing high quality fruit, the industry is threatened by CM, which was first introduced into South Africa in 1885 and has become established in the country since then [2,23]. Baculovirus products including Madex (Andermatt, Switzerland) and Carpovirusine (Arysta Lifescience), all formulated with CpGV-M, have been registered in South Africa and are used to control CM as part of an integrated pest management programme [24,25]. Alarm over reports of widespread resistance in field populations of CM to CpGV-M in Europe led to bioprospecting for endemic South African CpGV isolates [26]. Here, we report on the genome analysis of a novel CpGV isolate which was recovered from diseased larvae in the Free State Province of South Africa, where no CpGV products were previously applied. The potential of this isolate for management of resistance, should it occur in South Africa following intensive application of CpGV-M based biopesticides in the field, is discussed.

## 2. Materials and Methods

### 2.1. Genome Sequencing and Assembly

Genomic DNA was extracted from purified CpGV-SA occlusion bodies (OBs) recovered from field-collected larvae [26,27]. Approximately 100 ng of CpGV-SA genomic DNA was sequenced using a MiSeq Desktop Sequencer (Illumina, San Diego, CA, USA). Geneious R7 (Biomatters, Auckland, New Zealand) was used to conduct a *de novo* assembly, which produced 10,000 high quality contigs from 691,903 paired reads with an average coverage of 545.4 ± 191.3. The contigs were then mapped to the reference sequence, CpGV-M1, an in vivo cloned genotype of the Mexican isolate [28]. The consensus sequence generated was annotated using both CpGV-M1 and CpGV-M [6]. The assembled sequence of the CpGV-SA genome (NCBI GenBank: MN075941) was validated by comparing in silico and in vitro restriction endonuclease profiles [27]. The CpGV-SA genome was then used to detect single nucleotide variations (SNVs), within the isolate, and to analyse single nucleotide polymorphisms (SNPs) across geographically distinct isolates of CpGV.

### 2.2. Comparative Single Nucleotide Polymorphism Analysis

To identify SNPs (intra-species variation), a nucleotide alignment of CpGV-SA and CpGV-M, -I07, -I12, -S and -E2 complete genome sequences was first generated using ClustalW in Geneious R11. SNPs were identified by comparing each sequence against the others using the Variation/SNP tool in Geneious R11 with the minimum variant frequency set to 0.1 and effects on translations analysed. The resulting SNP data were exported to Excel 365 (Microsoft, Redmond, WA, USA) and analysed. SNPs were only considered for nucleotides in which all six sequences successfully aligned and were not disrupted by small insertions or deletions (Indel).

### 2.3. Genotypic Variation within the CpGV-SA Isolate

The above-mentioned raw Illumina data were reanalysed in Geneious R11 with paired reads error corrected and normalized using the BBNorm plugin. Reads were *de novo* assembled and the top 1000 contigs generated further, dissolved and mapped to the assembled CpGV-SA genome sequence. SNVs (variation within CpGV-SA) were detected amongst the aligned reads using the variation detection tool in Generous R11. A total of 972,303 reads, with Q20, Q30 and Q40 values of 91.0%, 82.9% and 39.6%, respectively, successfully mapped to the complete genome sequence. Data generated were exported to Excel 365 and analysed.

### 2.4. Phylogenetic Analysis

A new nucleotide alignment consisting of complete genome sequences from CpGV-SA, -M, -I07, -I12, -S, -E2 and Cryptophlebia leucotreta granulovirus (CrleGV) (NC_005068), as an outlier, was used to construct a minimum evolution (ME) phylogenetic tree. The phylogenetic tree was inferred by using 1000 bootstrap replicates with a close-neighbor-interchange algorithm with an initial tree obtained by neighbor joining. The analysis was conducted using MEGA X (Version 10) software [29].

## 3. Results

### 3.1. CpGV-SA Genome Characteristics

The complete genome sequence for CpGV-SA is 123,595 bp with coding regions of 110,551 bp (89.45%). The CpGV-SA genome sequence was initially annotated using the gene data from the reference sequence, CpGV-M1. However, the open reading frames (ORFs) of CpGV-M that were determined as different from CpGV-M1 were chosen as the annotations, due to having higher percentage identities when assessing the nucleotide and amino acid sequences. The genome has a 45.3% GC content and is comprised of 141 ORFs out of the 142 identified in CpGV-M. A total of 57 ORFs within the CpGV-SA genome are 100% identical to the corresponding ORFs in the CpGV-M isolate. ORFs cp25 and cp38 of CpGV-M1, encoded less than 50 amino acids (aa) and are therefore regarded as absent from the CpGV-SA genome [28,30].

Further analysis revealed that ORF cp24 (pe38) was truncated with a 24 bp deletion similar to CpGV-E2, -I07, -I12, and -S. Truncations were also observed in ORFs cp18, cp27, cp31, cp37 and cp62 by 112, 267, 91, 266 and 159 aa, respectively, when compared to CpGV-M. The truncation in ORFs 27 and 62 was particularly severe with approximately 60.6 and 83.6% of the coding region lost. Extended ORFs were identified for ORF cp6 and cp63 by 18 and 25 aa, respectively, in relation to CpGV-M. ORFs cp28/29, 50/51 and 129/130 were fused into single ORFs. Like most CpGV isolates, cp52 was split into two frames, cp52a and cp52b.

Several unique INDELs within ORFs were also identified in the CpGV-SA genome sequence. These include a 3 bp deletion in cp50/cp51 and cp102 at bases 42325 to 42327 and 85423 to 85425 in CpGV-M, respectively, with the former INDEL also present in CpGV-S and I07. Similarly, a deletion of 3 bp in cp115 was identified to disrupt the stop codon, located at positions 98349 to 98351 in CpGV-M, resulting in an extension of the ORF by 9 amino acids.

### 3.2. Comparative Single Nucleotide Polymorphism Analysis

A comparison of CpGV-SA against all other known CpGV isolates with complete genome sequences was conducted to identify novel SNPs and amino acid changes (Table 1). The pairwise identity of CpGV-SA to CpGV- E2, -I07, -I12, -M, and –S was 98.07%, 96.23%, 98.26%, 98.9% and 98.99%, respectively.

The comparison identified a total of 821 SNPs between CpGV-SA and CpGV-E2, -I07, -I12, -M, and –S, of which 67 were novel (Table 1). The SNPs resulted in a total of 352 amino acid substitutions amongst the various CpGV isolates, of which 24 were unique to CpGV-SA. Two polymorphisms were detected at position 98024 (97937 in CpGV-M shown in Table 1) with CpGV-SA and -E2 both differing from the other isolates.

### 3.3. Genotypic Variation within the CpGV-SA Isolate

Intra specific variation was analysed by reassembling and mapping of the Illumina reads to the complete CpGV-SA genome sequence described above. For this analysis, a mean coverage of 2210.7 (±911.4) per nucleotide was achieved with a minimum of 152 and maximum of 4438. A total of 66 genotypic SNVs were detected in the CpGV-SA read data when compared against the complete genome sequence (Table 2). The average quality was greater than Q30 for 95% for all reads utilized in detection of these SNVs. Of the 66 SNVs, 22 resulted in amino acid changes in protein sequences (Table 2).

Out of all 821 SNPs detected between the CpGV-SA and CpGV-M, I12, E2, S and I07 isolates, 31 were potentially due to genotypic variation within the South African isolate (highlighted light grey in Table 2). Of the 31 SNVs, 11 were identified to overlap with SNPs unique to CpGV-SA (highlighted dark grey in Table 2). For each of these, the genotypic variant identified matched the respective nucleotide present in the other CpGV isolates. The resulting number of unique SNPs present within the CpGV-SA isolate could therefore range between 56 and 67 depending on the genotypic mixture. The remaining 36 SNVs detected in CpGV-SA were not present in the SNP data. A single nucleotide, at position 51290 in the CpGV-SA genome, was detected to have three variants, either a T (as in the CpGV-SA reference sequence), an A or a C (marked * in Table 2).

Analysis of INDELs in CpGV-SA was also performed to identify variation within this and the genomes of the other CpGV isolates. Of the INDELs mentioned above, those occurring in ORF cp31, cp37 and cp62 of the CpGV-SA isolate, were absent amongst some of the genotypic variants resulting in the preservation of ORF as annotated in the other CpGV isolates. For example, CpGV-SA ORF cp62 was truncated due to the insertion of a single nucleotide at position 51479. However, this nucleotide was revealed to have considerable variation amongst the CpGV-SA Illumina data, with numerous reads indicating the absence of this insertion, resulting in an ORF like that of cp62 in the CpGV-S and E2 isolates. Similarly, other INDELs identified in CpGV-SA were found to match some which had been previously identified in other CpGV isolates resulting in similar ORFs. An example of this was CpGV-SA cp6, whereby an INDEL present within genotypic variants was identical to an ORF previously detected only in CpGV-S cp6.

### 3.4. Phylogenetic Analysis

**Figure 1:**
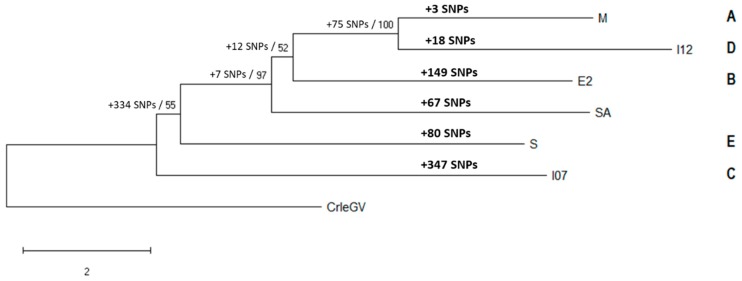
CpGV phylogeny based on complete genome sequences for isolates from groups A-E and CpGV-SA. The number of unique SNPs present in each isolate is shown above the branches in bold. The number of SNPs per grouping is shown at each node alongside the bootstrap values. CpGV groups are indicated to right of the respective isolate.

The phylogeny of the six CpGV isolates was determined by alignment and analysis of the complete genome sequences (Figure 1).

Phylogenetic analysis of the CpGV isolates placed the South African isolate basal to isolates M, I12 and E2 and distal to S and I07 with strong bootstrap support calculated (97%). Although low bootstrap support was calculated for the CpGV-E2, I07 and S isolates, all branches generated were dichotomous. CpGV-SA was also identified to have more unique SNPs (67) than the CpGV-M and I12 isolates (3 and 18, respectively).

## 4. Discussion

Initial characterization of CpGV-SA using restriction enzyme analysis of genomic DNA provided the first evidence that CpGV-SA is not only genetically different from other sequenced CpGV isolates, but also of mixed genotypes [27]. This observation prompted further in-depth genetic characterization by sequencing of the complete genome using the Illumina MiSeq platform, achieving an average coverage of 545.4 ± 191.3, considerably higher than that attained for the CpGV-M, I12, E2, S and I07 isolates [7]. This platform can generate large quantities of data with extremely low error rates of approximately 0.24% per base pair [31]. This not only enabled the comparative analysis of intra-species SNPs between CpGV-SA and other sequenced CpGV isolates, but also evaluation of genotypic variation or SNVs within the isolate.

Comparison of the CpGV-SA genome with other sequenced genomes identified a total of 821 SNPs compared to 788 reported previously by [7]. However, a comparison of the data generated in this study shows that only 526 of the 788 SNPs are still detectable. This difference is likely due to changes introduced during the alignment of all six genome sequences, with the inclusion of the South African isolate changing penalties incurred during gap openings and the degree to which these are extended. Therefore, instead of a direct comparison of the number of SNPs as previously reported, changes in SNP patterns, particularly those in ORFs, were evaluated. The analysis revealed that of the 664 SNPs identified between all six isolates, 67 were unique to CpGV-SA and the majority of these resulted in amino acid changes in protein sequences. Interestingly, phylogenetic analysis based on alignment of six CpGV complete genomes placed the CpGV-SA isolate basal to CpGV-M, I12 and E2 with no definite classification into any of the previously defined genogroups [5,6]. When combining the result of this tree with the SNP analysis and REN profiles [27], it appears that CpGV-SA is a genetic variant of previously described isolates and likely representative of a new phylogenetic group.

Analysis of intra species variation by reassembly and mapping of Illumina reads to the complete CpGV-SA genome sequence showed that, similar to previously analyzed CpGV isolates [5], the wild-type CpGV-SA isolate consists of a mixture of genotypes. Such a mixture of genotypes, as is found in natural populations of most wild-type baculovirus isolates [32,33], has important implications for the control of CM in South Africa where commercial formulations based on the Mexican isolate are extensively applied, risking the development of resistance in field CM populations as was the case in Europe. Notably, CpGV-SA has type I resistance breaking potential characteristic of other isolates currently registered in Europe and North America as biological control agents. Further studies are required to test the ability of CpGV-SA to overcome other types of resistance which, to date, is only achieved by the CpGV-E2 isolate [21,34].

In summary, the results of this study indicate that CpGV-SA is a unique isolate which is genetically distinct from those previously described and probably representative of a new genogroup. Although not exhibiting superior biological activity when compared to CpGV-M [26,27], the discovery of CpGV-SA may provide an additional valuable tool to enable alternating application of products based on diverse isolates for resistance management in South Africa and possibly in Europe where such resistance already occurs.

## Figures and Tables

**Table 1:** Alignment of 67 novel SNPs identified between CpGV-SA and CpGV-E2, -I07, -I12, -M, and –S, grouped as CpGV. Positions for each nucleotide are given relative to the CpGV-M and CpGV-SA sequences. The corresponding ORF numbers are provided above the alignment for SNPs occurring within coding regions while any resulting amino acid substitutions are shown below. Nucleotides: A = Adenine, C = Cytosine, G = Guanine, T = Thymine; Ambiguous nucleotides: Y = C or T and M = A or C and amino acids: A = Alanine, R = Arginine, N = Asparagine, Q = Glutamine, H = Histidine, I = Isoleucine, K = Lysine, P = Proline, S = Serine, T = Threonine, and V = Valine.

CpGV-M Position	1355	4732	6826	6909	7448	8659	12978	13476	13879	14318	15829	17138	17511	20146	20293	20314	20442	24975	27108	30837	34313	34485	38331	41720	43772	43925	51043	51049	51177	51398	51413	51483	51534	
CpGV-SA Position	1355	4785	6879	6962	7501	8712	13048	13546	13949	14389	15891	17199	17572	20185	20316	20337	20439	24946	27091	30847	34368	34540	38386	41775	43827	43980	51160	51166	51279	51308	51323	51393	51445	
ORF	3	7	10			11	17			18	20	22						30				41	46	50			61							
CpGV-SA	A	T	A	T	A	A	T	A	G	T	T	C	A	A	T	A	A	T	A	A	A	A	A	T	T	A	T	C	T	T	T	T	C	
CpGV	G	A	G	C	G	G	C	C	A	C	C	T	G	T	C	G	T	C	G	G	C	G	T	C	C	C	A	G	G	C	A	A	T	
Substitution SA -> Ref		K -> N	I -> T		S -> P						T -> A		T -> A					V -> A					I -> N			Q -> H	N -> I	R -> T						

CpGV-M Position	51537	58836	62466	64748	66714	67897	68732	70388	72378	72492	76189	77867	78942	84035	90782	93156	93414	94559	97937	98947	105867	106113	109603	110904	111093	111270	111535	115974	116322	116527	117053	120008	120642	122324
CpGV-SA Position	51448	58937	62567	64824	66790	67973	68808	70464	72454	72568	76265	77943	79018	84111	90852	93226	93484	94626	98024	99031	105933	106179	109669	110970	111158	111335	111600	116038	116386	116591	117117	120072	120706	122389
ORF		73	77	82	84	85	87	89	90		93	95	96	101		111		112	115			124			128			132		133	135	140		141
CpGV-SA	C	T	T	T	T	T	A	A	C	T	A	A	A	A	C	G	G	A	C	A	G	A	T	T	T	A	A	T	C	A	C	A	A	C
CpGV	T	C	G	C	C	C ^a^	C	G	T ^b^	C	G	G	G	G	T	C	A	T	G ^c^	G	A	G	A	C	C	G	G	C	T	G	T	C ^d^	C	T
Substitution SA -> Ref			E -> D				I -> R				N -> S			I -> T				Y -> F		T -> A			E -> V				K -> E	L -> S		V -> A	A -> T	I -> L	K -> T	A -> T
^a^ CpGV-E2 = Y; ^b^ CpGV = Y; ^c^ CpGV-E2 = A; ^d^ CpGV-E2 = M																																		

**Table 2:** Alignment of 66 genotypic SNVs identified within the CpGV-SA complete genome sequence. Variants which overlap with SNPs identified between the CpGV isolates are in light grey while those which overlap with SNPs unique to CpGV-SA (Table 1) are in dark grey. The corresponding ORF numbers are provided above the alignment for SNVs occurring within coding regions while any resulting amino acid substitutions are shown below. Nucleotides: A = Adenine, C = Cytosine, G = Guanine, T = Thymine and amino acids: A = Alanine, R = Arginine, N = Asparagine, D = Aspartic acid, E = Glutamic acid, G = Glycine, H = Histidine, I = Isoleucine, K = Lysine, M = Methionine, T = Threonine, Y = Tyrosine, and V = Valine.

CpGV-SA Position	6530	6823	6879	6962	7542	8502	8646	9003	9242	11275	11503	15891	17199	17572	20415	20439	20451	20471	27091	32302	34540	38386	40007	41403	43827	47598	47601	49694	51160	51166	51242	51259	51265
ORFs	10					11			12	15		20	22							37	41	46	48	50/51		57		60	61				
																									52b	58							
CpGV-SA	T	A	A	T	G	C	G	A	G	C	T	T	C	A	A	A	A	C	A	G	A	A	A	G	T	C	C	G	T	C	G	T	A
Variant	C	G	G	C	A	T	A	T	A	T	A	C	T	G	T	T	T	A	G	T	G	T	G	A	C	T	T	A	A	G	T	C	C
Variant Coverage	906	1452	1300	1210	642	1800	1172	1282	1338	584	474	1634	2158	794	750	2215	2201	1915	492	532	738	614	817	1544	840	1638	1388	840	2964	2782	1088	1426	1234
Amino Acid Change			I -> T		A -> V				A -> T	A -> T	N -> Y	T -> A		T -> A								I -> N	D -> G			A -> T	A -> T		N -> I	R -> T			

CpGV-SA Position	51273	51279	51284	51287	51289	51290*	51290*	51300	51301	51308	51323	51393	51445	51448	51605	62567	64981	66790	66971	70464	79018	82048	90574	90852	98024	99031	100326	111158	114455	117293	117676	119691	122389
ORFs																77	82b	84		89	96	99	110		115	116	117	128	131	135		139	141
CpGV-SA	C	T	A	T	C	**T**	**T**	G	T	T	T	T	C	C	T	T	T	T	C	A	A	G	G	C	C	A	A	T	T	T	C	G	C
Variant	T	G	T	G	A	**A**	**C**	C	A	C	A	A	T	T	A	G	C	C	T	G	G	A	A	T	G	G	G	C	C	C	T	A	T
Variant Coverage	1234	1266	1240	2136	3170	970	2420	1406	1390	3448	2756	3328	3422	3394	1392	1628	1742	880	462	1272	828	612	1724	1992	1630	1396	1224	904	544	1250	466	1492	1730
Amino Acid Change																E -> D						A -> V			I -> M	T -> A	K -> R		H -> R	D -> G		A -> V	A -> T
